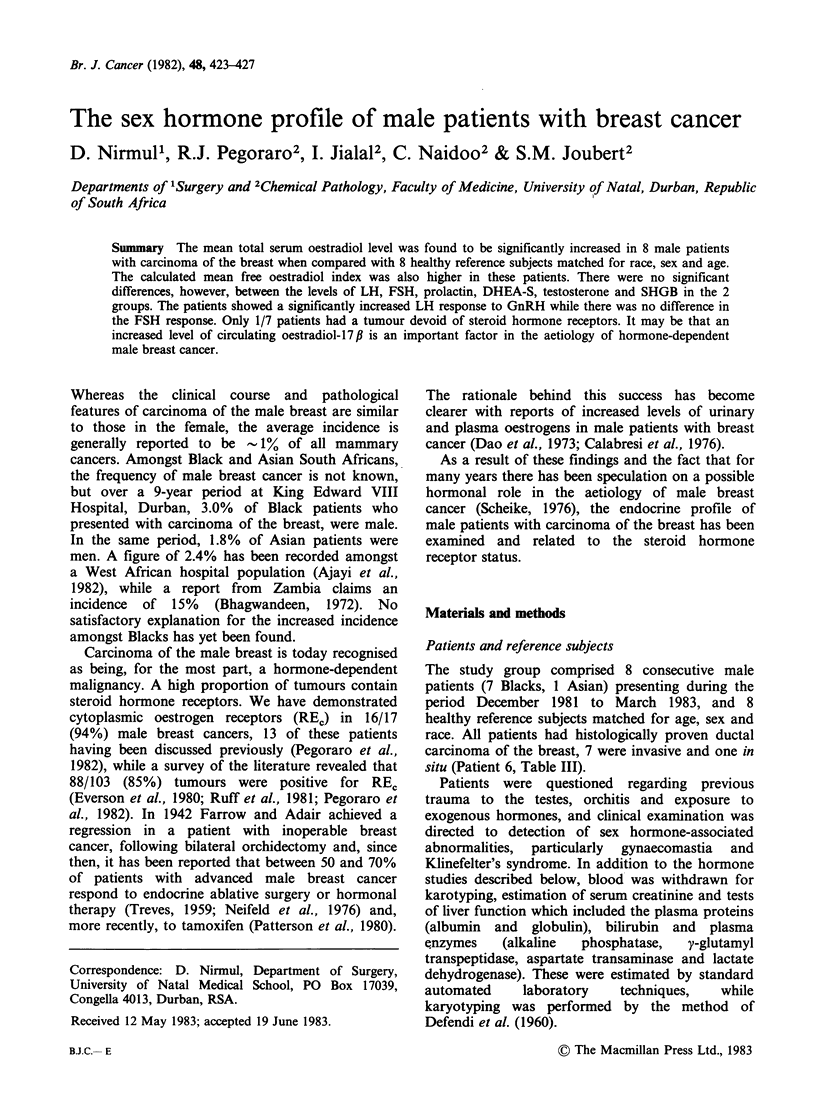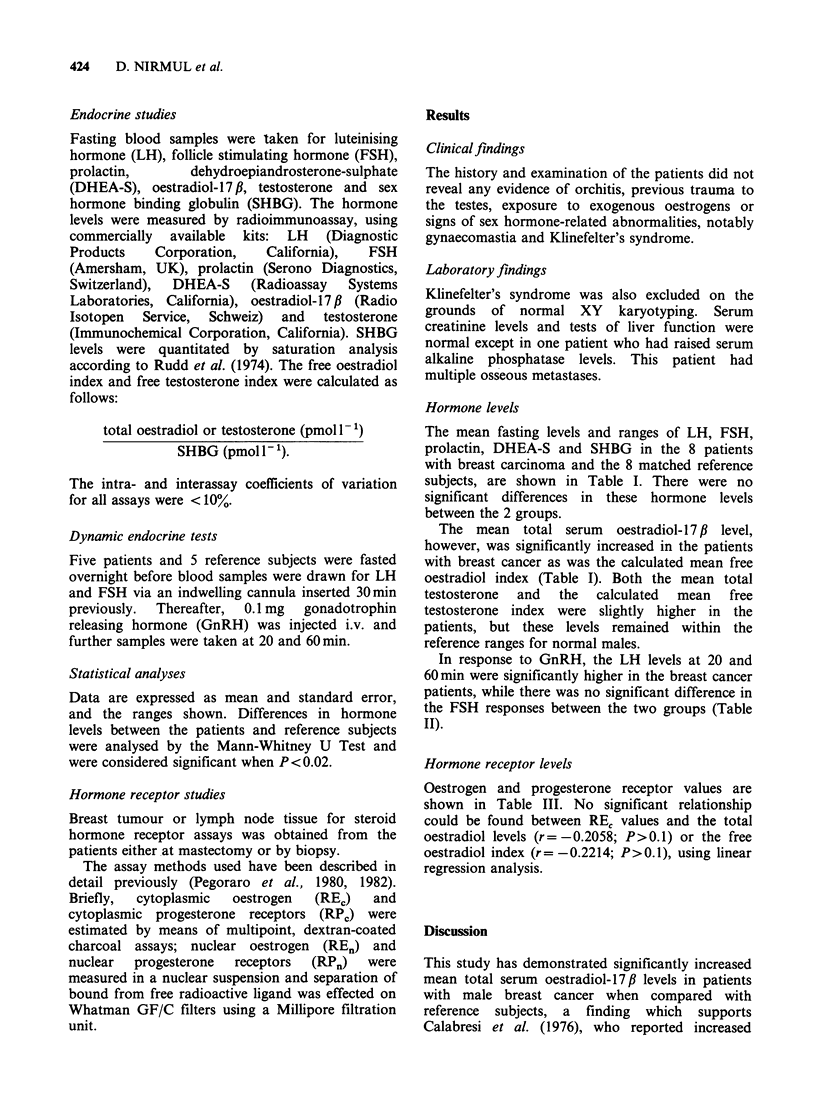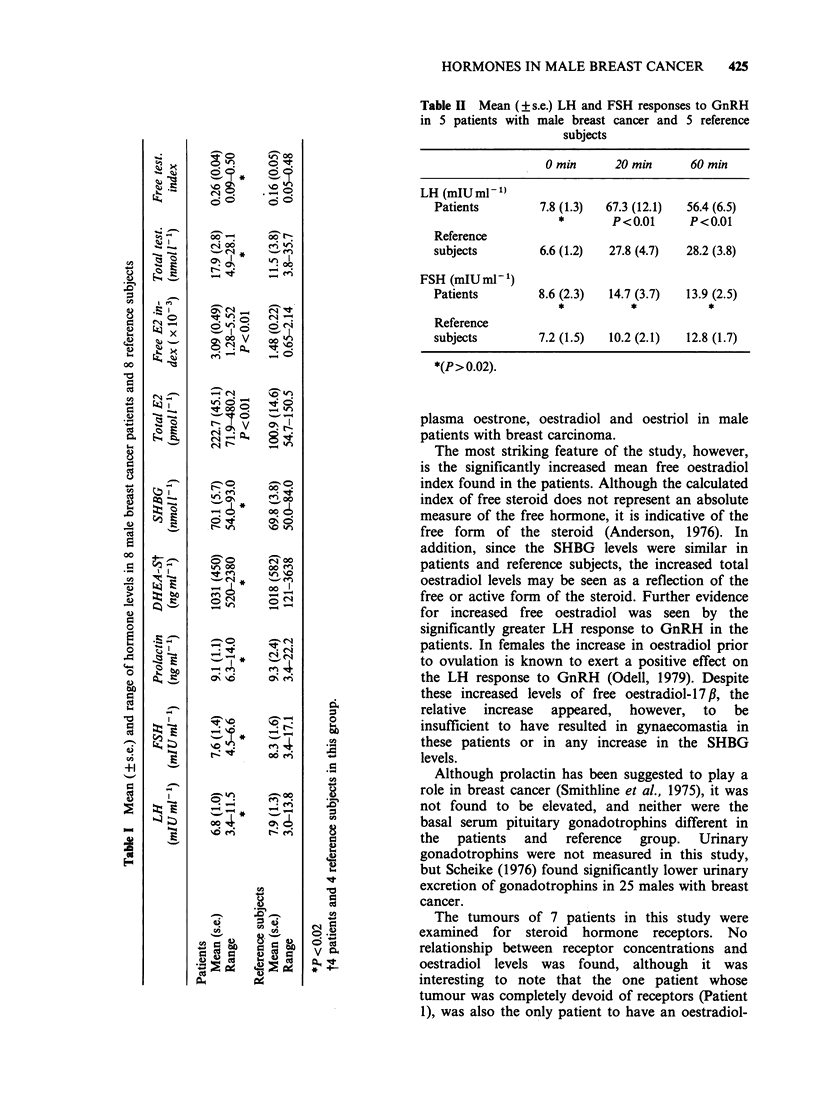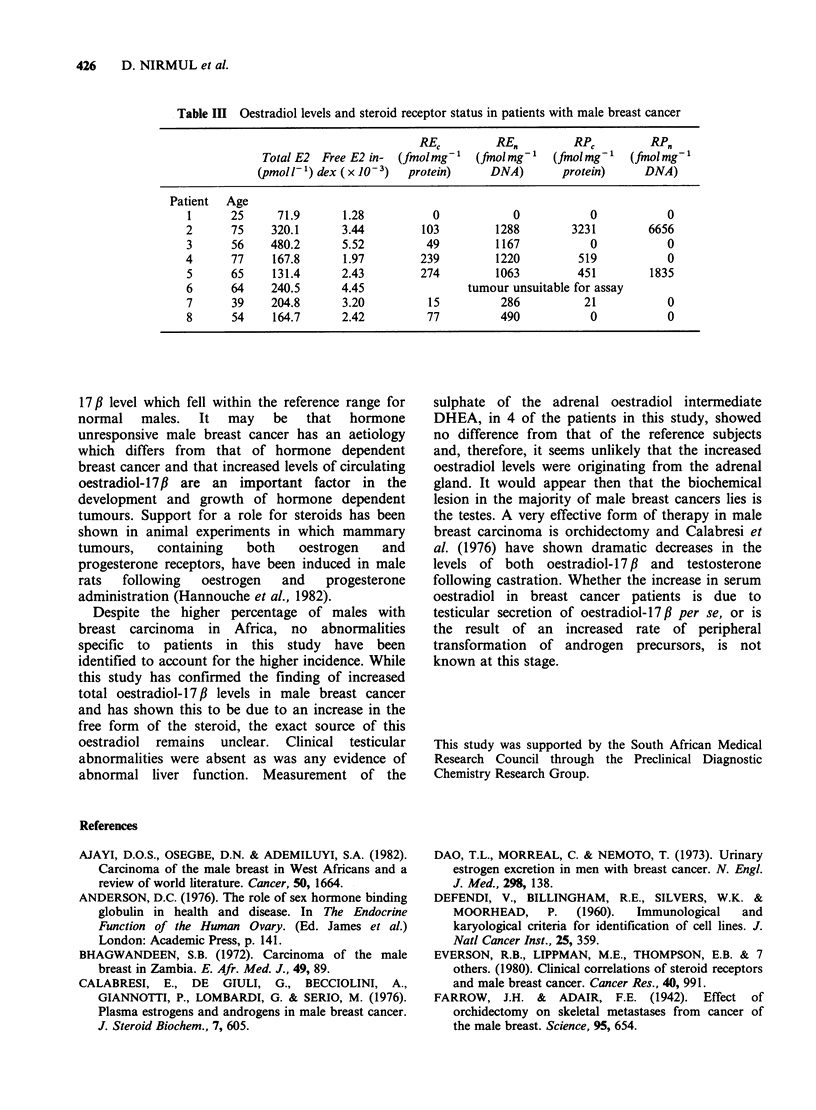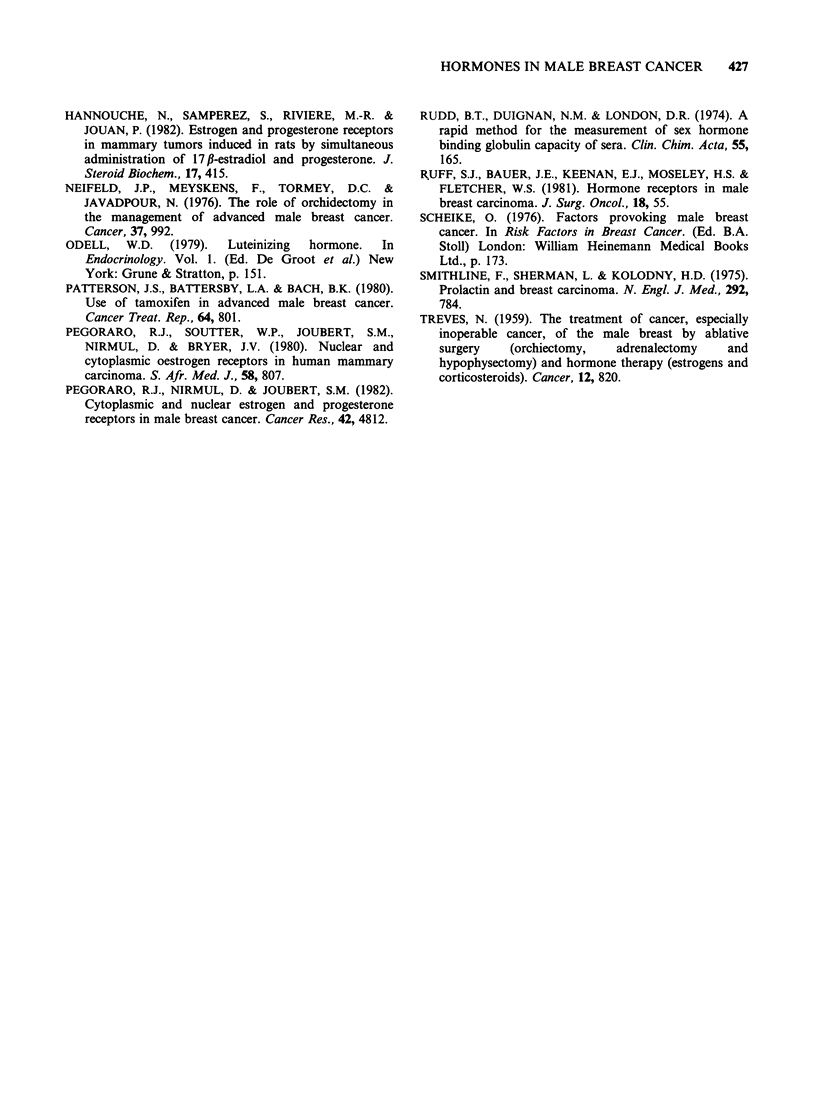# The sex hormone profile of male patients with breast cancer.

**DOI:** 10.1038/bjc.1983.208

**Published:** 1983-09

**Authors:** D. Nirmul, R. J. Pegoraro, I. Jialal, C. Naidoo, S. M. Joubert

## Abstract

The mean total serum oestradiol level was found to be significantly increased in 8 patients with carcinoma of the breast when compared with 8 healthy reference subjects matched for race, sex and age. The calculated mean free oestradiol index was also higher in these patients. There were no significant differences, however, between the levels of LH, FSH, prolactin. DHEA-S, testosterone and SHGB in the 2 groups. The patients showed a significantly increased LH response to GnRH while there was no difference in the FSH response. Only 1/7 patients had a tumour devoid of steroid hormone receptors. It may be that an increased level of circulating oestradiol-17 beta is an important factor in the aetiology of hormone-dependent male breast cancer.


*Br. J. Cancer* (1982), **48**, 423–427

### The sex hormone profile of male patients with breast cancer

D. Nirmul[1], R.J. Pegoraro[2], I. Jialal[2], C. Naidoo[2] & S.M. Joubert[2]

*Departments of [1]Surgery and [2]Chemical Pathology, Faculty of Medicine, University of Natal, Durban, Republic of South Africa*

**Summary** The mean total serum oestradiol level was found to be significantly increased in 8 male patients with carcinoma of the breast when compared with 8 healthy reference subjects matched for race, sex and age. The calculated mean free oestradiol index was also higher in these patients. There were no significant differences, however, between the levels of LH, FSH, prolactin, DHEA-S, testosterone and SHGB in the 2 groups. The patients showed a significantly increased LH response to GnRH while there was no difference in the FSH response. Only 1/7 patients had a tumour devoid of steroid hormone receptors. It may be that an increased level of circulating oestradiol-17$\beta$ is an important factor in the aetiology of hormone-dependent male breast cancer.

Whereas the clinical course and pathological features of carcinoma of the male breast are similar to those in the female, the average incidence is generally reported to be $\sim 1\%$ of all mammary cancers. Amongst Black and Asian South Africans, the frequency of male breast cancer is not known, but over a 9-year period at King Edward VIII Hospital, Durban, 3.0% of Black patients who presented with carcinoma of the breast, were male. In the same period, 1.8% of Asian patients were men. A figure of 2.4% has been recorded amongst a West African hospital population (Ajayi *et al.*, 1982), while a report from Zambia claims an incidence of 15% (Bhagwandeen, 1972). No satisfactory explanation for the increased incidence amongst Blacks has yet been found.

Carcinoma of the male breast is today recognised as being, for the most part, a hormone-dependent malignancy. A high proportion of tumours contain steroid hormone receptors. We have demonstrated cytoplasmic oestrogen receptors ($RE_c$) in 16/17 (94%) male breast cancers, 13 of these patients having been discussed previously (Pegoraro *et al.*, 1982), while a survey of the literature revealed that 88/103 (85%) tumours were positive for $RE_c$ (Everson *et al.*, 1980; Ruff *et al.*, 1981; Pegoraro *et al.*, 1982). In 1942 Farrow and Adair achieved a regression in a patient with inoperable breast cancer, following bilateral orchidectomy and, since then, it has been reported that between 50 and 70% of patients with advanced male breast cancer respond to endocrine ablative surgery or hormonal therapy (Treves, 1959; Neifeld *et al.*, 1976) and, more recently, to tamoxifen (Patterson *et al.*, 1980).

Correspondence: D. Nirmul, Department of Surgery, University of Natal Medical School, PO Box 17039, Congella 4013, Durban, RSA.

Received 12 May 1983; accepted 19 June 1983.

The rationale behind this success has become clearer with reports of increased levels of urinary and plasma oestrogens in male patients with breast cancer (Dao *et al.*, 1973; Calabresi *et al.*, 1976).

As a result of these findings and the fact that for many years there has been speculation on a possible hormonal role in the aetiology of male breast cancer (Scheike, 1976), the endocrine profile of male patients with carcinoma of the breast has been examined and related to the steroid hormone receptor status.

### Materials and methods

### *Patients and reference subjects*

The study group comprised 8 consecutive male patients (7 Blacks, 1 Asian) presenting during the period December 1981 to March 1983, and 8 healthy reference subjects matched for age, sex and race. All patients had histologically proven ductal carcinoma of the breast, 7 were invasive and one *in situ* (Patient 6, Table III).

Patients were questioned regarding previous trauma to the testes, orchitis and exposure to exogenous hormones, and clinical examination was directed to detection of sex hormone-associated abnormalities, particularly gynaecomastia and Klinefelter's syndrome. In addition to the hormone studies described below, blood was withdrawn for karotyping, estimation of serum creatinine and tests of liver function which included the plasma proteins (albumin and globulin), bilirubin and plasma enzymes (alkaline phosphatase, $\gamma$-glutamyl transpeptidase, aspartate transaminase and lactate dehydrogenase). These were estimated by standard automated laboratory techniques, while karyotyping was performed by the method of Defendi *et al.* (1960).

© The Macmillan Press Ltd., 1983

### Endocrine studies

Fasting blood samples were taken for luteinising hormone (LH), follicle stimulating hormone (FSH), prolactin, dehydroepiandrosterone-sulphate (DHEA-S), oestradiol-17$\beta$, testosterone and sex hormone binding globulin (SHBG). The hormone levels were measured by radioimmunoassay, using commercially available kits: LH (Diagnostic Products Corporation, California), FSH (Amersham, UK), prolactin (Serono Diagnostics, Switzerland), DHEA-S (Radioassay Systems Laboratories, California), oestradiol-17$\beta$ (Radio Isotopen Service, Schweiz) and testosterone (Immunochemical Corporation, California). SHBG levels were quantitated by saturation analysis according to Rudd *et al.* (1974). The free oestradiol index and free testosterone index were calculated as follows:

$$\frac{\text{total oestradiol or testosterone (pmol l}^{-1}\text{)}}{\text{SHBG (pmol l}^{-1}\text{)}}.$$

The intra- and interassay coefficients of variation for all assays were $<10\%$.

### Dynamic endocrine tests

Five patients and 5 reference subjects were fasted overnight before blood samples were drawn for LH and FSH via an indwelling cannula inserted 30 min previously. Thereafter, 0.1 mg gonadotrophin releasing hormone (GnRH) was injected i.v. and further samples were taken at 20 and 60 min.

### Statistical analyses

Data are expressed as mean and standard error, and the ranges shown. Differences in hormone levels between the patients and reference subjects were analysed by the Mann-Whitney U Test and were considered significant when $P<0.02$.

### Hormone receptor studies

Breast tumour or lymph node tissue for steroid hormone receptor assays was obtained from the patients either at mastectomy or by biopsy.

The assay methods used have been described in detail previously (Pegoraro *et al.*, 1980, 1982). Briefly, cytoplasmic oestrogen ($RE_c$) and cytoplasmic progesterone receptors ($RP_c$) were estimated by means of multipoint, dextran-coated charcoal assays; nuclear oestrogen ($RE_n$) and nuclear progesterone receptors ($RP_n$) were measured in a nuclear suspension and separation of bound from free radioactive ligand was effected on Whatman GF/C filters using a Millipore filtration unit.

### Results

### Clinical findings

The history and examination of the patients did not reveal any evidence of orchitis, previous trauma to the testes, exposure to exogenous oestrogens or signs of sex hormone-related abnormalities, notably gynaecomastia and Klinefelter's syndrome.

### Laboratory findings

Klinefelter's syndrome was also excluded on the grounds of normal XY karyotyping. Serum creatinine levels and tests of liver function were normal except in one patient who had raised serum alkaline phosphatase levels. This patient had multiple osseous metastases.

### Hormone levels

The mean fasting levels and ranges of LH, FSH, prolactin, DHEA-S and SHBG in the 8 patients with breast carcinoma and the 8 matched reference subjects, are shown in Table I. There were no significant differences in these hormone levels between the 2 groups.

The mean total serum oestradiol-17$\beta$ level, however, was significantly increased in the patients with breast cancer as was the calculated mean free oestradiol index (Table I). Both the mean total testosterone and the calculated mean free testosterone index were slightly higher in the patients, but these levels remained within the reference ranges for normal males.

In response to GnRH, the LH levels at 20 and 60 min were significantly higher in the breast cancer patients, while there was no significant difference in the FSH responses between the two groups (Table II).

### Hormone receptor levels

Oestrogen and progesterone receptor values are shown in Table III. No significant relationship could be found between $RE_c$ values and the total oestradiol levels ($r=-0.2058$; $P>0.1$) or the free oestradiol index ($r=-0.2214$; $P>0.1$), using linear regression analysis.

### Discussion

This study has demonstrated significantly increased mean total serum oestradiol-17$\beta$ levels in patients with male breast cancer when compared with reference subjects, a finding which supports Calabresi *et al.* (1976), who reported increased

**Table I** Mean (±s.e.) and range of hormone levels in 8 male breast cancer patients and 8 reference subjects

| | *LH* (*mIU ml*$^{-1}$) | *FSH* (*mIU ml*$^{-1}$) | *Prolactin* (*ng ml*$^{-1}$) | *DHEA-S*† (*ng ml*$^{-1}$) | *SHBG* (*nmol l*$^{-1}$) | *Total E2* (*pmol l*$^{-1}$) | *Free E2 index* ($\times 10^{-3}$) | *Total test.* (*nmol l*$^{-1}$) | *Free test. index* |
|---|---|---|---|---|---|---|---|---|---|
| Patients | | | | | | | | | |
| Mean (s.e.) | 6.8 (1.0) | 7.6 (1.4) | 9.1 (1.1) | 1031 (450) | 70.1 (5.7) | 222.7 (45.1) | 3.09 (0.49) | 17.9 (2.8) | 0.26 (0.04) |
| Range | 3.4–11.5 | 4.5–6.6 | 6.3–14.0 | 520–2380 | 54.0–93.0 | 71.9–480.2 | 1.28–5.52 | 4.9–28.1 | 0.09–0.50 |
| | * | * | * | * | * | $P<0.01$ | $P<0.01$ | * | * |
| Reference subjects | | | | | | | | | |
| Mean (s.e.) | 7.9 (1.3) | 8.3 (1.6) | 9.3 (2.4) | 1018 (582) | 69.8 (3.8) | 100.9 (14.6) | 1.48 (0.22) | 11.5 (3.8) | 0.16 (0.05) |
| Range | 3.0–13.8 | 3.4–17.1 | 3.4–22.2 | 121–3638 | 50.0–84.0 | 54.7–150.5 | 0.65–2.14 | 3.8–35.7 | 0.05–0.48 |

*$P<0.02$

†4 patients and 4 reference subjects in this group.

**Table II** Mean (±s.e.) LH and FSH responses to GnRH in 5 patients with male breast cancer and 5 reference subjects

| | *0 min* | *20 min* | *60 min* |
|---|---|---|---|
| LH (mIU ml$^{-1}$) | | | |
| Patients | 7.8 (1.3) | 67.3 (12.1) | 56.4 (6.5) |
| | * | $P<0.01$ | $P<0.01$ |
| Reference subjects | 6.6 (1.2) | 27.8 (4.7) | 28.2 (3.8) |
| FSH (mIU ml$^{-1}$) | | | |
| Patients | 8.6 (2.3) | 14.7 (3.7) | 13.9 (2.5) |
| | * | * | * |
| Reference subjects | 7.2 (1.5) | 10.2 (2.1) | 12.8 (1.7) |

*($P>0.02$).

plasma oestrone, oestradiol and oestriol in male patients with breast carcinoma.

The most striking feature of the study, however, is the significantly increased mean free oestradiol index found in the patients. Although the calculated index of free steroid does not represent an absolute measure of the free hormone, it is indicative of the free form of the steroid (Anderson, 1976). In addition, since the SHBG levels were similar in patients and reference subjects, the increased total oestradiol levels may be seen as a reflection of the free or active form of the steroid. Further evidence for increased free oestradiol was seen by the significantly greater LH response to GnRH in the patients. In females the increase in oestradiol prior to ovulation is known to exert a positive effect on the LH response to GnRH (Odell, 1979). Despite these increased levels of free oestradiol-17$\beta$, the relative increase appeared, however, to be insufficient to have resulted in gynaecomastia in these patients or in any increase in the SHBG levels.

Although prolactin has been suggested to play a role in breast cancer (Smithline *et al.*, 1975), it was not found to be elevated, and neither were the basal serum pituitary gonadotrophins different in the patients and reference group. Urinary gonadotrophins were not measured in this study, but Scheike (1976) found significantly lower urinary excretion of gonadotrophins in 25 males with breast cancer.

The tumours of 7 patients in this study were examined for steroid hormone receptors. No relationship between receptor concentrations and oestradiol levels was found, although it was interesting to note that the one patient whose tumour was completely devoid of receptors (Patient 1), was also the only patient to have an oestradiol-

**Table III** Oestradiol levels and steroid receptor status in patients with male breast cancer

| Patient | Age | *Total E2 (pmol $l^{-1}$)* | *Free E2 index ($\times 10^{-3}$)* | *$RE_c$ (fmol $mg^{-1}$ protein)* | *$RE_n$ (fmol $mg^{-1}$ DNA)* | *$RP_c$ (fmol $mg^{-1}$ protein)* | *$RP_n$ (fmol $mg^{-1}$ DNA)* |
|---|---|---|---|---|---|---|---|
| 1 | 25 | 71.9 | 1.28 | 0 | 0 | 0 | 0 |
| 2 | 75 | 320.1 | 3.44 | 103 | 1288 | 3231 | 6656 |
| 3 | 56 | 480.2 | 5.52 | 49 | 1167 | 0 | 0 |
| 4 | 77 | 167.8 | 1.97 | 239 | 1220 | 519 | 0 |
| 5 | 65 | 131.4 | 2.43 | 274 | 1063 | 451 | 1835 |
| 6 | 64 | 240.5 | 4.45 | | tumour unsuitable for assay | | |
| 7 | 39 | 204.8 | 3.20 | 15 | 286 | 21 | 0 |
| 8 | 54 | 164.7 | 2.42 | 77 | 490 | 0 | 0 |

17*β* level which fell within the reference range for normal males. It may be that hormone unresponsive male breast cancer has an aetiology which differs from that of hormone dependent breast cancer and that increased levels of circulating oestradiol-17*β* are an important factor in the development and growth of hormone dependent tumours. Support for a role for steroids has been shown in animal experiments in which mammary tumours, containing both oestrogen and progesterone receptors, have been induced in male rats following oestrogen and progesterone administration (Hannouche *et al.*, 1982).

Despite the higher percentage of males with breast carcinoma in Africa, no abnormalities specific to patients in this study have been identified to account for the higher incidence. While this study has confirmed the finding of increased total oestradiol-17*β* levels in male breast cancer and has shown this to be due to an increase in the free form of the steroid, the exact source of this oestradiol remains unclear. Clinical testicular abnormalities were absent as was any evidence of abnormal liver function. Measurement of the sulphate of the adrenal oestradiol intermediate DHEA, in 4 of the patients in this study, showed no difference from that of the reference subjects and, therefore, it seems unlikely that the increased oestradiol levels were originating from the adrenal gland. It would appear then that the biochemical lesion in the majority of male breast cancers lies is the testes. A very effective form of therapy in male breast carcinoma is orchidectomy and Calabresi *et al.* (1976) have shown dramatic decreases in the levels of both oestradiol-17*β* and testosterone following castration. Whether the increase in serum oestradiol in breast cancer patients is due to testicular secretion of oestradiol-17*β* *per se,* or is the result of an increased rate of peripheral transformation of androgen precursors, is not known at this stage.

This study was supported by the South African Medical Research Council through the Preclinical Diagnostic Chemistry Research Group.

### References

AJAYI, D.O.S., OSEGBE, D.N. & ADEMILUYI, S.A. (1982). Carcinoma of the male breast in West Africans and a review of world literature. *Cancer,* **50,** 1664.

ANDERSON, D.C. (1976). The role of sex hormone binding globulin in health and disease. In *The Endocrine Function of the Human Ovary.* (Ed. James *et al.*) London: Academic Press, p. 141.

BHAGWANDEEN, S.B. (1972). Carcinoma of the male breast in Zambia. *E. Afr. Med. J.,* **49,** 89.

CALABRESI, E., DE GIULI, G., BECCIOLINI, A., GIANNOTTI, P., LOMBARDI, G. & SERIO, M. (1976). Plasma estrogens and androgens in male breast cancer. *J. Steroid Biochem.,* **7,** 605.

DAO, T.L., MORREAL, C. & NEMOTO, T. (1973). Urinary estrogen excretion in men with breast cancer. *N. Engl. J. Med.,* **298,** 138.

DEFENDI, V., BILLINGHAM, R.E., SILVERS, W.K. & MOORHEAD, P. (1960). Immunological and karyological criteria for identification of cell lines. *J. Natl Cancer Inst.,* **25,** 359.

EVERSON, R.B., LIPPMAN, M.E., THOMPSON, E.B. & 7 others. (1980). Clinical correlations of steroid receptors and male breast cancer. *Cancer Res.,* **40,** 991.

FARROW, J.H. & ADAIR, F.E. (1942). Effect of orchidectomy on skeletal metastases from cancer of the male breast. *Science,* **95,** 654.

HANNOUCHE, N., SAMPEREZ, S., RIVIERE, M.-R. & JOUAN, P. (1982). Estrogen and progesterone receptors in mammary tumors induced in rats by simultaneous administration of 17$\beta$-estradiol and progesterone. *J. Steroid Biochem.*, **17,** 415.

NEIFELD, J.P., MEYSKENS, F., TORMEY, D.C. & JAVADPOUR, N. (1976). The role of orchidectomy in the management of advanced male breast cancer. *Cancer,* **37,** 992.

ODELL, W.D. (1979). Luteinizing hormone. In *Endocrinology*. Vol. 1. (Ed. De Groot *et al.*) New York: Grune & Stratton, p. 151.

PATTERSON, J.S., BATTERSBY, L.A. & BACH, B.K. (1980). Use of tamoxifen in advanced male breast cancer. *Cancer Treat. Rep.*, **64,** 801.

PEGORARO, R.J., SOUTTER, W.P., JOUBERT, S.M., NIRMUL, D. & BRYER, J.V. (1980). Nuclear and cytoplasmic oestrogen receptors in human mammary carcinoma. *S. Afr. Med. J.*, **58,** 807.

PEGORARO, R.J., NIRMUL, D. & JOUBERT, S.M. (1982). Cytoplasmic and nuclear estrogen and progesterone receptors in male breast cancer. *Cancer Res.*, **42,** 4812.

RUDD, B.T., DUIGNAN, N.M. & LONDON, D.R. (1974). A rapid method for the measurement of sex hormone binding globulin capacity of sera. *Clin. Chim. Acta,* **55,** 165.

RUFF, S.J., BAUER, J.E., KEENAN, E.J., MOSELEY, H.S. & FLETCHER, W.S. (1981). Hormone receptors in male breast carcinoma. *J. Surg. Oncol.*, **18,** 55.

SCHEIKE, O. (1976). Factors provoking male breast cancer. In *Risk Factors in Breast Cancer*. (Ed. B.A. Stoll) London: William Heinemann Medical Books Ltd., p. 173.

SMITHLINE, F., SHERMAN, L. & KOLODNY, H.D. (1975). Prolactin and breast carcinoma. *N. Engl. J. Med.*, **292,** 784.

TREVES, N. (1959). The treatment of cancer, especially inoperable cancer, of the male breast by ablative surgery (orchiectomy, adrenalectomy and hypophysectomy) and hormone therapy (estrogens and corticosteroids). *Cancer,* **12,** 820.